# Selective synthesis of Fe_3_O_4_Au_*x*_Ag_*y*_ nanomaterials and their potential applications in catalysis and nanomedicine

**DOI:** 10.1186/s13065-017-0288-y

**Published:** 2017-06-24

**Authors:** Essy Kouadio Fodjo, Koffi Mouroufié Gabriel, Brou Yapi Serge, Dan Li, Cong Kong, Albert Trokourey

**Affiliations:** 1 Laboratory of Physical Chemistry, Université Felix Houphouet-Boigny, 22 BP 582, Abidjan 22, Côte d’Ivoire; 2 Institut National Polytechnique Felix Houphouet-Boigny, BP 1093, Yamoussoukro, Côte d’Ivoire; 3 School of Chemical and Environmental Engineering, Shanghai Institute of Technology, Shanghai, 201418 People’s Republic of China; 4 East China Sea Fisheries Research Institute, Chinese Academy of Fishery Sciences, No. 300, Jungong Road, Yangpu, Shanghai, 200090 People’s Republic of China

**Keywords:** Magnetite-based nanoparticles, Synthesis and application of nanoparticles, Core–shell nanoparticles, Magnetic resonance imaging, Drug delivery

## Abstract

In these recent years, magnetite (Fe_3_O_4_) has witnessed a growing interest in the scientific community as a potential material in various fields of application namely in catalysis, biosensing, hyperthermia treatments, magnetic resonance imaging (MRI) contrast agents and drug delivery. Their unique properties such as metal–insulator phase transitions, superconductivity, low Curie temperature, and magnetoresistance make magnetite special and need further investigation. On the other hand, nanoparticles especially gold nanoparticles (Au NPs) exhibit striking features that are not observed in the bulk counterparts. For instance, the mentioned ferromagnetism in Au NPs coated with protective agents such as dodecane thiol, in addition to their aptitude to be used in near-infrared (NIR) light sensitivity and their high adsorptive ability in tumor cell, make them useful in nanomedicine application. Besides, silver nanoparticles (Ag NPs) are known as an antimicrobial agent. Put together, the ${\text{Fe}}_{ 3} {\text{O}}_{ 4} {\text{Au}}_{x} {\text{Ag}}_{y} \left( {\left\{ {x,y} \right\} = \{ 0 , 1\} } \right)$ nanocomposites with tunable size can therefore display important demanding properties for diverse applications. In this review, we try to examine the new trend of magnetite-based nanomaterial synthesis and their application in catalysis and nanomedicine.

## Background

**Fig. 1:**
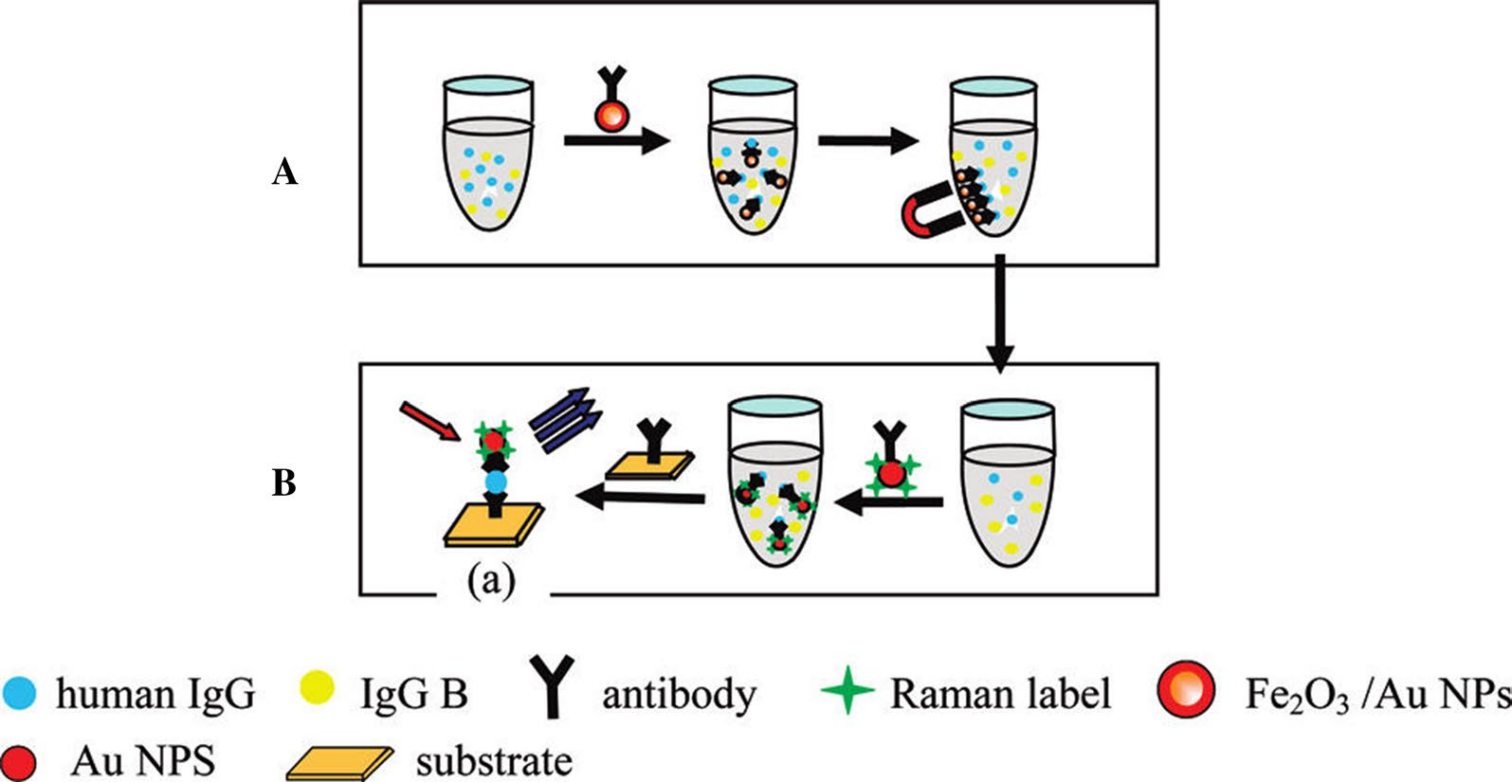
**A** Antigens separation by Fe_2_O_3_/Au core/shell nanoparticles, and **B** subsequent rapid detection by immunoassay analysis based on SERS [6]

Nanostructures have inherited particular properties which are linked with their size and their morphology. These physical properties have a significant effect on their application [1, 2]. Among these nanostructures which have aroused a huge application, magnetic iron oxide (Fe_3_O_4_ and Fe_2_O_3_) NPs have attracted much attention especially in the catalysis for chemical degradation and biomedical applications due to their low toxicity, superparamagnetic and low Curie temperature. Indeed, in recent studies, magnetite nanocomposites have been successfully used as a magnetically recyclable catalyst for the degradation of organic compounds [3, 4] while further research [5] have demonstrated the non-toxicity of the Fe_3_O_4_ nanoparticles on rat mesenchymal stem cells and their ability to label the cells. However, these iron oxides are unstable due to their ability to undergo oxidation easily [5]. To overcome this issue, a combination with noble metal NPs such as silver (Ag) or gold (Au) has been used. This combination provides not only an important stability for these iron oxides in solution, but also the ability to bind various biological ligands with convenient enhancement of optical and magnetic properties (Fig. 1) [6–8]. These Fe_3_O_4_Au_*x*_Ag_*y*_ NPs have the advantages to be useful in suspension application. Such a suspension can interact with an external magnetic field to facilitate a magnetic separation or can be guided to a specific area, thus facilitating a magnetic resonance imaging for medical diagnosis and an alternating current (AC) magnetic field-assisted cancer therapy [9–11].

Furthermore, in the core–shell types, the properties of the nanostructures change from one structure to another, depending on the size, the shape and the shell or core composition. In this nanostructure, the shell can prevent the core from corrosion or dissolution. This effect can lead to a major enhancement of the thermal, mechanical, and electrical properties of the system [12, 13]. Besides, because of the coupling between the spectrally localized surface plasmon resonance (LSPR) of the noble metal NPs and the continuum of interband transitions of the other hybrid component in the core–shell nanostructures, Fano resonances (FR) in strongly coupled systems can arise. This plasmon hybridization can be rationalized and can serve as a good substitute in biological applications [14, 15].

Since the dimensions of the individual components are nanoscale level or comparable to the size of the biomolecules, the combination is always expected to proffer novel functions which are not available in single-component materials. However, only appropriate sizes of the Fe_3_O_4_Au_*x*_Ag_*y*_ NPs exhibit such properties and are superparamagnetic [16]. Therefore, tailoring of an appropriate nanostructure constitutes the main problem.

Moreover, in a recent review, Ali et al. [17]. have examined a large range of synthesis methods of nanomaterials. In these methods, mechanochemical (i.e., laser ablation arc discharge, combustion, electrodeposition, and pyrolysis) and chemical (sol–gel synthesis, template-assisted synthesis, reverse micelle, hydrothermal, co-precipitation, etc.) methods have extensively been studied. According to these authors, various shapes and size of NPs (i.e., nanorod, porous spheres, nanohusk, nanocubes, distorted cubes, and self-oriented flowers) can be obtained using nearly matching synthetic protocols by simply changing the reaction parameters [18]. Although they claimed the possibility to synthesize specific size and shape, they did not show the different routes to produce these physical properties. In this review, we will intensively discuss the way to design Fe_3_O_4_Au_*x*_Ag_*y*_ namely Fe_3_O_4_ nanocomposites in which Au and Ag are involved. Particular interest will be paid to core–shell nanostructures and their application in catalysis and nanomedicine.

### Synthesis methods

#### Fe_3_O_4_ synthesis

Among the most popular synthesis methods, co-precipitation is widely used for the synthesis of Fe_3_O_4_ NPs. It is convenient and considered as the easiest method. In this method, Fe^2+^ and Fe^3+^ are the main precursor in solution. The starting molar ratio Fe^2+^/Fe^3+^ [19, 20], the basicity (NaOH, NH_4_OH, and CH_3_NH_2_) [21], and the ionic strength [N(CH_3_)_4_^+^, CH_3_NH_3_^+^, NH_4_^+^, Na^+^, Li^+^, and K^+^] [22, 23] of the media play a major role. For instance, studies performed by Laurent et al. [24] have shown a change in magnetite NPs size by adjusting the basicity and the ion strength, and a change in shape by tuning the electrostatic surface density of the nanoparticles. For Patsula et al. [5], the synthesis of different shape, size, and particle size distribution of Fe_3_O_4_ can be done through the different reaction temperatures, the concentration of the stabilizer, and the type of high-boiling-point solvents. Other factors such as an inlet of nitrogen gas or agitation are also critical in achieving the desired size, and the morphology of the magnetite NPs [25].

Moreover, in base media for instance, Fe(OH)_2_ and Fe(OH)_3_ are easily formed. The aqueous mixture of Fe^2+^ and Fe^3+^ sources at Fe^3+^/Fe^2+^ = 2:1 molar ratio can lead to a black color product of Fe_3_O_4_ [26] which is governed by Eq. (1):

1 $${\text{Fe}}^{ 2+ } + 2 {\text{Fe}}^{ 3+ } + 8 {\text{OH}}^{ - } \to {\text{Fe}}_{ 3} {\text{O}}_{ 4} + 4 {\text{H}}_{ 2} {\text{O}}$$

In recent study [27], it has been reported that the molar ratios smaller than Fe^3+^/Fe^2+^ = 2:1 cannot compensate the oxidation of Fe^2+^ to Fe^3+^ for the preparation of Fe_3_O_4_ nanoparticles under oxidizing environment. However, in synthesis evolving in anaerobic conditions, a complete precipitation of Fe_3_O_4_ is likely formed, and no attentiveness is needed about the starting Fe^3+^/Fe^2+^ ratio as the excess of Fe^2+^ can be converted into Fe^3+^ in the Fe_3_O_4_ lattice as described by Schikorr reaction (Eq. 2):

2 $$3{\text{Fe}}\left( {\text{OH}} \right)_{2} \to {\text{Fe}}_{3} {\text{O}}_{4} + {\text{H}}_{2} + 2{\text{H}}_{2} {\text{O}}$$

At low temperature, with the presence of organic compounds, the anaerobic conditions can also give rise to the formation of “green rust”. Likely, the excess of iron(II) hydroxide in the medium along with this green rust can progressively be transformed into iron(II, III) oxide. It should also be noted that all along these syntheses in aqueous media, the pH of the reaction mixture has to be adjusted in the synthesis and the purification steps to achieve smaller monodisperse NPs. Furthermore, in an oxygen-free environment, most preferably in the presence of N_2_, the bubbling nitrogen gas can help to prevent the NPs from oxidation or to reduce the size of the NPs [16].

#### Synthesis of Fe_3_O_4_Au_*x*_Ag_*y*_

The hybrid nanostructures with two or more components have attracted more attention due to the synergistic properties induced by their interactions. In the synthesis of nanocomposites, several techniques such as co-reduction of mixed ions, organic-phase temporary linker and seed-mediated growth have been explored [28, 29]. All of them have proven their feasibility and advantages. The aim of the application is the main motivation of the chosen technique as the structure and surface composition of the shell or the core are among the primordial parameters on which the properties of the nanocomposites are subjugated [30, 31].

The co-reduction of mixed ions is known to be less selective in core–shell synthesis. In this procedure, the component which acts as the core can be formed randomly depending on the reactions parameters (pH, temperature, agitation, duration of the reaction, standard potential associated with each ion, etc.) [32, 33]. Furthermore, when designing a solution-based synthetic system for core/shell multicomponent nanocrystals, it is important to consider the electronegativity of the metals for the selection of the appropriate reducing agent. It is relatively difficult to judge whether they can be prepared in a designed synthetic system because of their huge difference in the oxidizing power. This electronegativity is important to avoid polydispersity and keep the by-products in nanoscale level. This process is not suitable for Fe_3_O_4_ nanocomposites synthesis as the iron ions may undergo reduction, but it can be used for the Au–Ag nanocomposite synthesis.

**Fig. 2:**
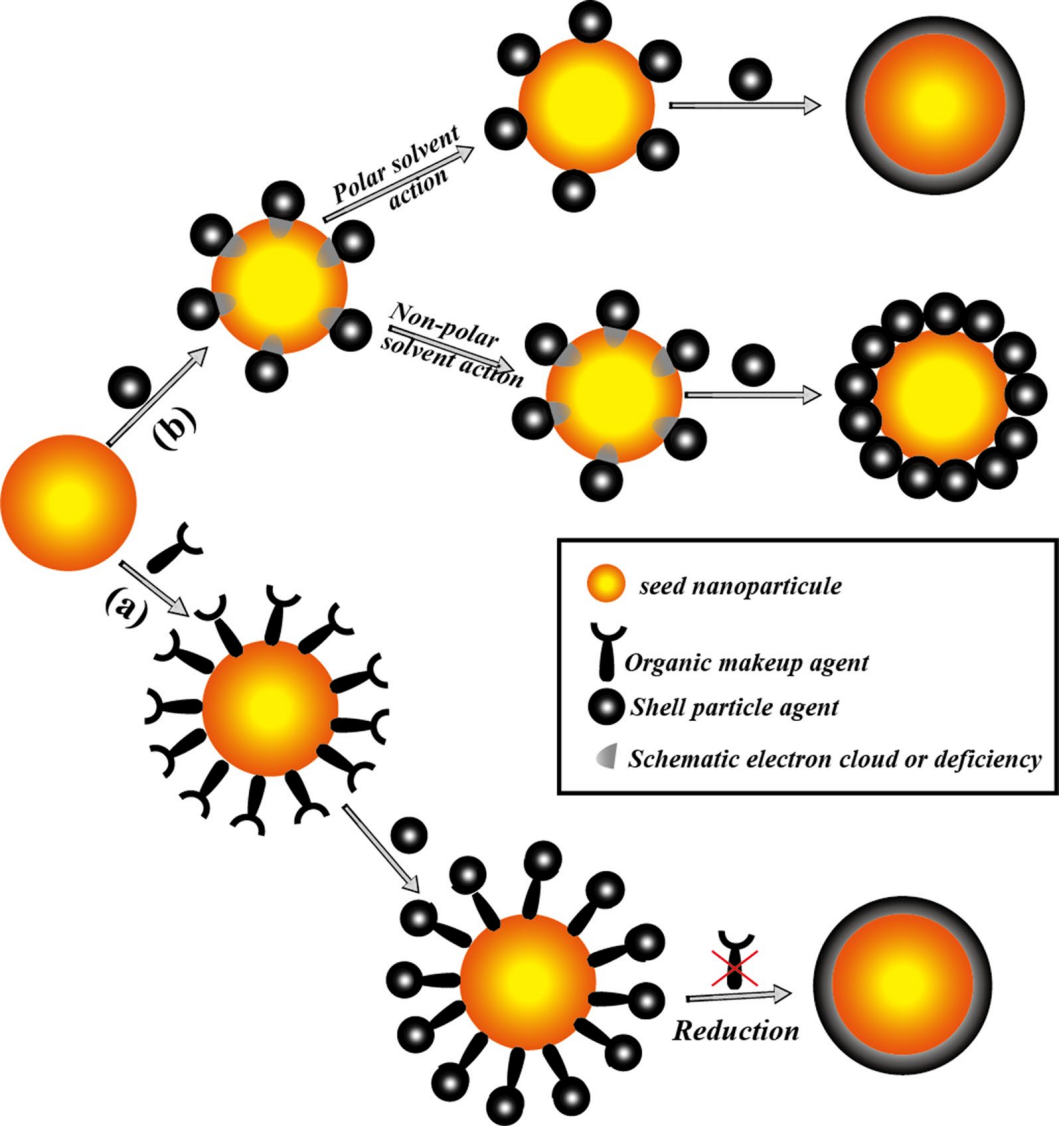
Schematic diagram showing the mechanism of formation of core/shell NPs and heterodimers: (*a*) chemical makeup method and (*b*) seed-mediated technique

As the properties depend on the component which acts as core or shell, an appropriate design can be achieved using typical synthetic route to avoid the haphazard core/shell formation. For instance, for a given application, one would want to have a selected component as the core. This aim can be achieved efficiently using chemical makeup (Fig. 2a) of functional groups (organic-phase temporary linker) to modify the selected core surface. In this purpose, hydrophilic functional groups such as NH_2_ and SH can promote the attachment of the selected metal as a core while hydrophobic functional groups such as CH_3_ and PPh_2_ lead to minimal attachment [8, 34]. In this process, the adsorption of one component onto the core is affected by the surface charge, the solution pH, and the precursor concentrations. The thickness of the shell and the size of the core are strongly pH-dependent [35–37]. This chemical makeup method can also be used to prevent iron NPs from oxidation and agglomeration [23].

Another similar method without chemical makeup is seed-mediated growth (Fig. 2b). In this process, the core/shell NPs is designed by growing a uniform shell on the core NPs through adsorption of the shell compound ions on the seed-mediated NPs [38, 39]. This growth technique can be used for the fabrication of NPs such as Fe_3_O_4_Au_*x*_Ag_*y*_ with controlled size by acting on the precursor concentration of the shell component. In addition, the seed particles themselves can participate in the reaction as catalysts, where charge transfer between the seeds and newly nucleated components is involved. This effect lowers the energy for heterogeneous nucleation. As long as the reactant concentration, seed-to-precursor ratio, and heating profile are controlled, core/shell nanostructures or multicomponent heterostructures [40] and the desired thickness [41] can be obtained.

**Fig. 3:**
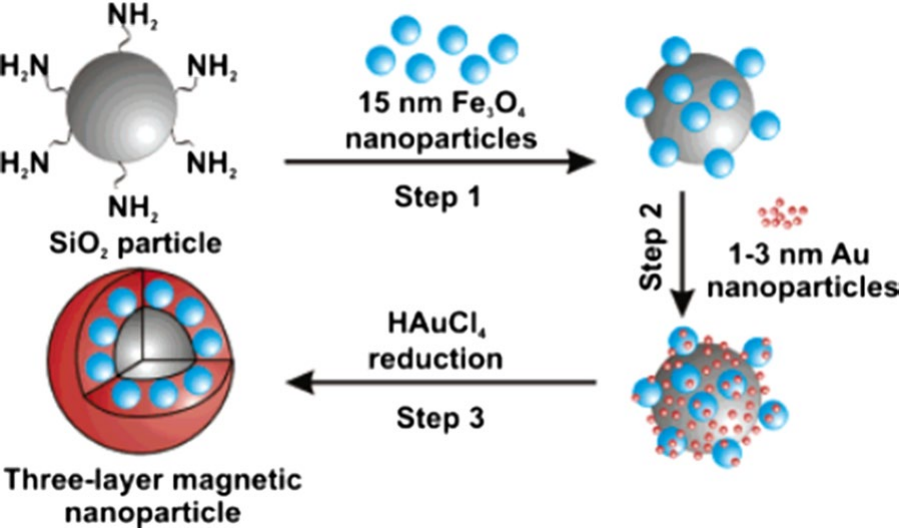
Synthetic scheme for the preparation of heterodimer nanoparticles by chemical makeup (*Step 1*) method and seed-mediated technique (*Step 3*) [46]

Besides core–shell nanostructures, heteromultimers with two joined NPs (Fig. 3, Step 1 and 2) sharing a common interface can be synthesized. The growth of heteromultimers follows procedures similar to those of core–shell NPs synthesis methods. However, a convenient route to control core/shell vs. heterodimer formation is mainly obtained by controlling the polarity of the solvent (Fig. 2b). It has been proposed that when a magnetic component, such as Fe_3_O_4_, is nucleated on Au or Ag, electrons will transfer from Au or Ag to Fe_3_O_4_ through the interface to match their chemical potentials [42, 43]. The charge transfer leads to electron deficiency on the metal (Au or Ag). If a polar solvent is used in the reaction, the electron deficiency on the metal can be replenished from the solvent leading therefore to the formation of multiple nucleation sites. This process results in continuous shell formation. On the other hand, if a non-polar solvent is used, once a single nucleated site depletes the electrons from the metal, the electron deficiency cannot be replenished from the solvent. This phenomenon prevents new nucleation events and promotes heterodimer NPs [44–46]. Additionally, strong reductant such as sodium borohydride promotes heterodimer formation [47].

### Physicochemical properties of Fe_3_O_4_ and nanocomposites

**Fig. 4:**
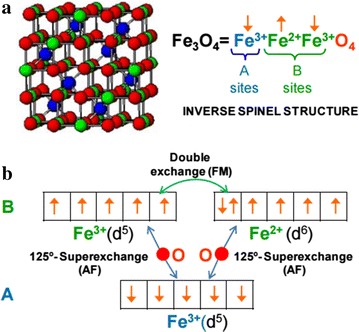
**a** The inverse spinel structure of Fe_3_O_4_, consisting of an FCC oxygen lattice, with tetrahedral (*A*) and octahedral (*B*) site. **b** Scheme of the exchange interaction in magnetite [50]

Fe_3_O_4_ is a typical magnetic iron oxide with a cubic inverse spinel structure in which oxygen forms a face-centered cubic close packing. Some of the Fe^3+^ occupy 1/8th of interstitial tetrahedral while equal amounts of Fe^3+^ and Fe^2+^ fill half of the available octahedral sites [48, 49] in the space group $Fd3\bar{m}$ with 0.8394 nm as lattice parameter. The conduction and the magnetism properties are mainly due to the distribution of these iron ions. Indeed, the electron spins of the Fe^2+^ and Fe^3+^ in the octahedral sites are coupled while the spins of the Fe^3+^ in the tetrahedral sites are anti-parallel coupled to those in octahedral sites. As the magnetic moments of the Fe^3+^ and Fe^2+^ are 5 and 4 Bohr magneton respectively, it results a magnetic moment equal to (→5←4←5) = 4 Bohr magneton (Fig. 4). This net effect is that the magnetic contributions of both sets are not balanced and it raises a permanent magnetism.

Because of a double-exchange interaction existing between Fe^2+^ and Fe^3+^ in octahedral sites due to d orbital overlap between iron atoms, the additional spin-down electron can hop between neighboring octahedral-sites, thereby resulting in a high conductivity [20, 50]. The electrical conductivity in Fe_3_O_4_ is generally caused by the superposition of the surface plasmon (SP) band and SP hopping conduction. Indeed, below room temperature, the band conduction is the dominant transport mechanism [51–53]. However, these properties can be improved when magnetite NPs are doped using a specific component such as Au and Ag. The obtained nanostructures can easily and promptly be induced into magnetic resonance by self-heating, applying the external magnetic field, or by moving along the attraction field [25, 54, 55]. Owing to the quantized oscillation of conduction electrons under an external electromagnetic field, these NPs can exhibit strong surface plasmon resonance (SPR) absorption similar to the metal NPs themselves [56, 57]. The ideal core size to obtain a perfect SPR is around 10 nm, and when this size is far less, these NPs show little or no SPR absorption [58, 59]. The controlled coating of either Au or Ag on the Fe_3_O_4_/(Au, Ag) NPs facilitates the tuning of the plasmonic properties of these core/shell NPs. Moreover, depositing a thicker Au shell on the magnetite NPs leads to a red-shift of the absorption band, while coating Ag on these seed particles results in a blue-shift of the absorption band compared with the metal absorption band itself. These phenomena are relevant to the shell thickness and the metal polarization regarding some parameters such as the dielectric environments, the refractive index of the second component or the charge repartition on the metal [40].

### Applications

#### Fe_3_O_4_Au_*x*_Ag_*y*_ catalytic properties

**Fig. 5:**
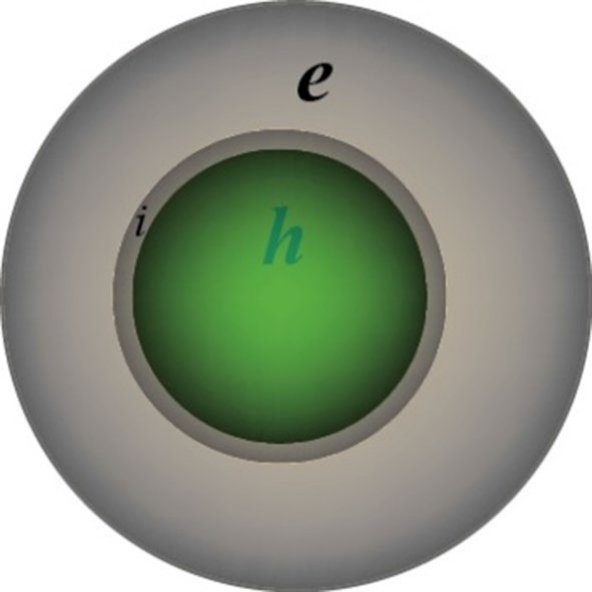
Arbitrary charge separation in core–shell nanostructures: (*i*) interface, (*e*) high density of electron and (*h*) high density of hole

The physicochemical properties of Fe_3_O_4_Au_*x*_Ag_*y*_ arise from the polarization effect at the interfaces of the different component of Fe_3_O_4_Au_*x*_Ag_*y*_. This polarization allows Fe_3_O_4_Au_*x*_Ag_*y*_ hybrid nanostructures to form a storage structure of electrons (Fig. 5) which are discharged when exposed to an electron acceptor such as O_2_, or organic compound. This structure can therefore give the ability of displaying a high catalytic activity towards an electron-transfer reaction, or excellent surface-enhanced Raman scattering activity when Au or Ag acts as a shell [60–62]. The formation of a space-charge layer at two different components interface is known to improve charge separation under band gap excitation, thus generating high density catalytic hot pot sites [63]. Indeed, these nanocomposites are useful in promoting light induced electron-transfer reactions and can be used as a powerful material for charge separation. In addition, individually, Ag NPs have a high antibacterial activity [64], Au NPs are optical active [65] while Fe_3_O_4_ NPs are supermagnetic [9]. All these properties make Fe_3_O_4_Au_*x*_Ag_*y*_ NPs convenient to be used in magnetic separation and in catalytic degradation of pollutants. Another advantage is that they are perfectly recycled in several folds [66].

#### Nanomedicine application

Avoiding alteration of healthy cell in chemotherapy, immunotherapy and radiotherapy is a major concern as these techniques do not specifically target the cancerous cells [67]. In a recent study [41], the toxicity grade of magnetic NPs on mouse fibroblast cell line has been classified as grade 1, which belongs to no cytotoxicity. Besides, the hemolysis rates are found to be far less than 5% while an acute toxicity testing in beagle dogs has shown no significant difference in body weight and no behavioral changes. Meanwhile, blood parameters, autopsy, and histopathological studies have shown no significant difference compared with those of the control group. These results suggest that Fe_3_O_4_Au_*x*_Ag_*y*_ NPs can be considered as an alternative agent to overcome the observed side effects in tumor treatment.

However, the trend in Fe_3_O_4_Au_*x*_Ag_*y*_ NPs concept is to deliver the drugs such as anticancer and at the same time, to observe what happens to the cancerous cells without damaging the healthy cell. This concept can be achieved thanks to the antimicrobial, magnetic and optic activities of the Fe_3_O_4_Au_*x*_Ag_*y*_ NPs. These hybrid NPs can be ideally used as magnetic resonance imaging (MRI) contrast enhancement agents.

**Fig. 6:**
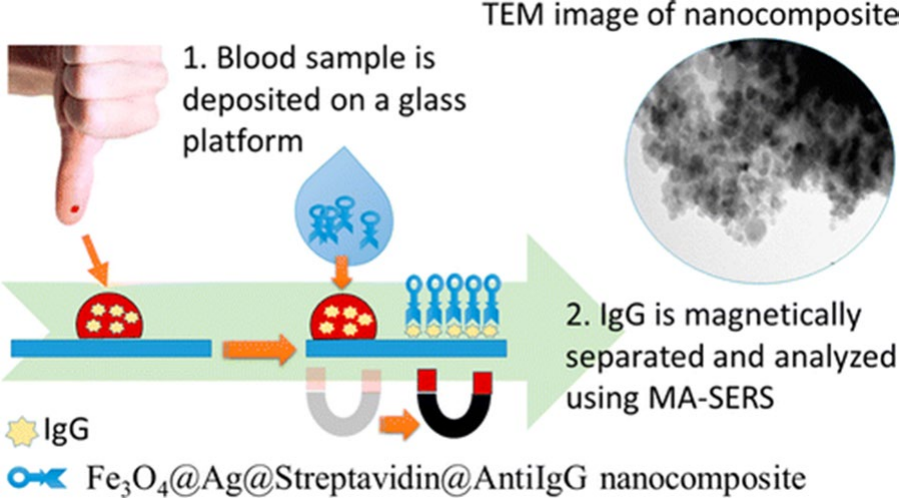
Determination of human immunoglobulin G using a novel approach based on magnetically (Fe_3_O_4_@Ag) assisted surface enhanced Raman spectroscopy [68]

In recent studies [6, 45, 68–72], authors have also shown that Fe_3_O_4_Au_*x*_Ag_*y*_ NPs can be manipulated using external magnetic field either for a magnetic separation of biological products (Fig. 6), a magnetic field-assisted cancer therapy and site-specific drug delivery or as a magnetic guidance of particle systems for MRI and for surface enhanced Raman spectroscopy detection.

Well-engineered Fe_3_O_4_Au_*x*_Ag_*y*_ NPs can effectively guide heat to the tumor without damaging the healthy tissue as injected Fe_3_O_4_Au_*x*_Ag_*y*_ nano-sized particles tend to accumulate in the tumor. This accumulation is done either passively through the enhanced permeability and retention effect or actively through their conjugation with a targeted molecule due to the unorganized nature of its vasculature. When applying hyperthermia with these NPs, the tumor temperature can increase up to 45 °C whereas the body temperature remains at around 38 °C [73, 74]. This ability of such NPs prevents the healthy cell from being altered.

In addition, gold NPs are known to be strong near-infrared (NIR) absorbers. Their effectiveness in cancer like breast and tumor optical contrast has been demonstrated and, the optical contrast of the tumor can be increased by 1 ~ 3.5 dB using injected Au NPs [75]. The applications of Fe_3_O_4_Au_*x*_Ag_*y*_ NPs have therefore not only the magnetism properties of iron oxide that renders them to be easily manipulated and heated by an external magnetic field, but also an excellent NIR light sensitivity and a high adsorptive ability from the metal layer which make them useful for photothermal therapy [41, 76, 77].

## Conclusions

Recent synthetic efforts have led to the understanding of the formation of a large variety of multicomponent NPs with different levels of complexity. A selected nanostructure with hybrid components can be synthesized by tailoring the synthesis parameters. Despite these exciting new developments, the study of multicomponent NPs is still at its infant stage compared with most single-element systems. In this purpose the mastery of the synthesis process of Fe_3_O_4_Au_*x*_Ag_*y*_ nanocomposites may be a milestone for their extensive application. Furthermore, the strong coupling between the different components exhibits novel physical phenomena and enhance their properties, thus, making them superior to their single-component counterparts for their application in nanomedicine and catalysis. This novel agent will help in diagnosis and treatment of terminal diseases efficiently by using their guiding capability. They may also provide an alternative to the highly toxic chemotherapy or thermotherapy, with the use of less toxic nano-carriers as anticancer agents and with less heat for healthy cells.

This application may pave a new dimension in cancer treatment and management in the near future. Another benefit of Fe_3_O_4_Au_*x*_Ag_*y*_ nanocomposites may be found in their highly catalytic properties for contaminant degradation in industry and waste processing. This last point is imperative for fighting against upstream roots of water-borne diseases.

## Abbreviations

AC: alternating current
FR: Fano resonances
LSPR: localized surface plasmon resonance
MRI: magnetic resonance imaging
NPs: nanoparticles
NIR: near-infrared
